# RUNX oncoproteins and miRNA networks

**DOI:** 10.18632/oncotarget.20673

**Published:** 2017-09-07

**Authors:** James C. Neil, Jodie Hay, Gillian Borland

**Affiliations:** Molecular Oncology Laboratory, MRC University of Glasgow Centre for Virus Research, University of Glasgow, Bearsden, Glasgow, UK

**Keywords:** RUNX1/AML1, oncoprotein, translocation, miRNA, sponge

The three mammalian RUNX genes have evolved distinctive and highly lineage-specific roles in development but show evidence of redundant functions in oncogenesis where they may function either as oncogenes or tumor suppressors according to context [1]. The core binding complexes formed by RUNX proteins and their cofactor CBFB accomplish their essential roles by activating or repressing target gene expression, and these processes are regulated by post-translational modification and interaction with cofactors. Not surprisingly, RUNX regulation also engages the miRNA network [2], as RUNX transcriptional regulation orchestrates the expression of specific miRNAs, while the expression of the RUNX proteins is in turn modulated by miRNA activity. Much of this control is exerted through miRNA target sites in the RUNX 3′UTRs that are unusually long and divergent in sequence, strongly suggesting a role for miRNA regulation in lineage-specific aspects of RUNX function.

The RUNX1(AML1) gene first came to prominence in the blood cancer field due to its frequent involvement in human leukemia where it is subject to a diverse array of chromosomal translocations, of which the archetype and most common is the t(8;21) translocation found in many cases of acute myeloid leukemia (AML). This translocation results in the expression of a fusion protein with an N-terminus derived from RUNX1, including the Runt DNA binding domain but lacking the C-terminal regulatory domain. The truncated RUNX1 is fused to the C-terminal moiety of ETO, a zinc finger protein that serves to recruit co-repressors to target genes [3]. The RUNX1-ETO complex appears to contribute to AML development mainly by blocking terminal differentiation. While early studies suggested that the fusion protein may be a constitutive and dominant negative inhibitor of all RUNX targets, more recent studies have shown that AML cell viability requires the continued expression of the wild-type RUNX1 protein from the un-translocated allele as well as the RUNX1-ETO fusion protein [4]. It appears that leukemic cell survival and growth depends on a critical balance between RUNX1 and its fusion oncoprotein derivatives.

A general feature of fusion oncoproteins is that they assemble modular protein functional domains from heterologous proteins to create products that are foreign to the cellular regulatory apparatus. Moreover, they are expressed from hybrid mRNAs in which the 3′UTR is attached to a new 5′ moiety, creating a novel structure that is likely to perturb the miRNA regulatory network. An interesting example of this phenomenon and its importance for leukemia development is provided by the recent paper from Zaidi in Oncotarget [5] where the authors show that the tumor suppressive miR-29b-1 forms a negative feedback loop with the RUNX1-ETO fusion protein. This miRNA was first described as a target for positive regulation by RUNX3 in gastric cancer cells [6] and appears to be down-regulated in t(8;21) cell lines where the promoter is bound by RUNX1-ETO and co-repressors. Significantly, ectopic expression of miR-29b-1 inhibits cell growth and promotes both apoptosis and terminal differentiation [5].

**Figure 1:**
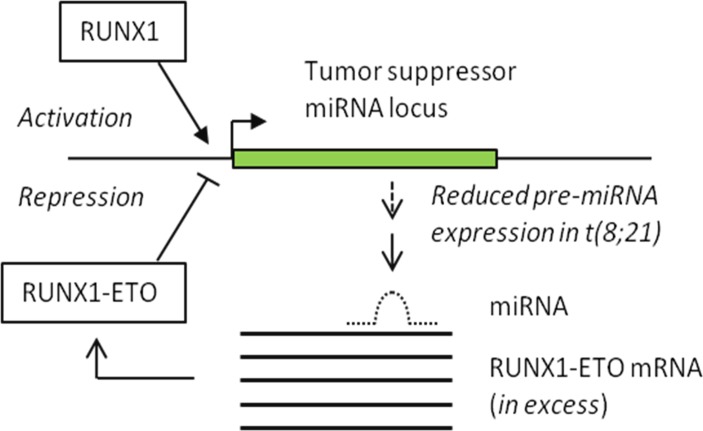
The figure depicts a model for the subversion of miRNA regulation due to the t(8;21) translocation in AML and expression of the RUNX1-ETO fusion oncoprotein. RUNX1 and RUNX1-ETO respectively activate and repress transcription of tumor suppressor premiRNAs exemplified by miR-29b-1 (ref 5). The effect of RUNX1-ETO repression may be reinforced by overexpression of the 3′ UTR of ETO under control of the RUNX1 promoter, creating a sponge for miR-29b-1 and other tumor suppressive miRNAs (ref 7).

These new findings provoke further questions about the dynamics of gene regulation and protein translation. For example, the accumulation of RUNX1-ETO protein in AML cells argues that the demonstrated miR-29b-1 negative feedback through its 3′UTR is not fully dominant, even though RUNX1 is also detectable on its promoter and is presumably driving expression at some level in AML cells [5]. This scenario invites consideration of the recent prediction that the t(8;21)-specific over-expression of the ETO 3′UTR under the control of the RUNX1 promoter creates a miRNA sponge [7]. According to this model, by its presence in molar excess the sponge overwhelms the negative regulatory activity of miR-29b-1 and other miRNAs with tumor suppressive potential. Moreover, as illustrated this process could operate synergistically with direct transcriptional repression of miRNAs by RUNX1-ETO (see Figure 1).

Studies of end-stage t(8;21) leukemias are revealing an intricate landscape of genetic and epigenetic changes [8]. Post-transcriptional changes involving the miRNA apparatus adds a further dimension to this complex picture. Moreover, the t(8;21) translocation often arises *in utero* long before the appearance of AML and requires secondary mutations to reveal its full oncogenic potential in model systems [3]. If new broadly effective therapies are to be generated it will be important to distinguish the direct effects of RUNX1-ETO such as those described [5] from the stochastic events that drive leukemic cell expansion and progression.
